# The effects of aging on semen parameters and sperm DNA fragmentation

**DOI:** 10.5935/1518-0557.20190058

**Published:** 2020

**Authors:** Víctor Pino, Antonia Sanz, Nicolás Valdés, Javier Crosby, Antonio Mackenna

**Affiliations:** 1 Faculty of Medicine, University of Los Andes, Santiago, Chile.; 2 Environmental Chemist, Department of Epidemiology, Faculty of Obstetrics and Medicine, University of Los Andes, Santiago, Chile.; 3 Biologist and Doctorate in Science, Unit of Reproductive Medicine, Department of Obstetrics and Gynecology, Clínica Las Condes, Santiago, Chile.; 4 Unit of Reproductive Medicine, Department of Obstetrics and Gynecology, Clínica Las Condes, Santiago, Chile.

**Keywords:** fertility, infertility, paternal age, aging, semen analysis, sperm DNA fragmentation

## Abstract

**Introduction:**

This study aimed to look into possible correlations between male age and different sperm parameters derived from semen analysis and sperm deoxyribonucleic acid (DNA) fragmentation.

**Methods:**

This retrospective descriptive study included 2681 male patients who underwent semen analysis at Clínica Las Condes (CLC), Santiago, Chile, between January 2014 and May 2017; correlations between age and sperm parameters were analyzed.

**Results:**

Males above the age of 50 were significantly more likely to present anomalies in semen volume, sperm concentration, and sperm DNA fragmentation; males aged 41+ years were more likely to have lower sperm concentration levels; males aged 31+ years were more likely to have decreased sperm motility; when concentration was constant, more volume and motility anomalies were seen as age increased; when volume was kept constant, more motility and concentration anomalies were seen as age increased; and when motility was constant, normal semen volumes decreased as age increased.

**Conclusion:**

Our study showed that male age significantly affects sperm parameters that might have an impact on male fertility.

## INTRODUCTION

Aging is a natural inevitable process that affects every individual and introduces a series of physiological changes in our bodies. One of the changes associated with aging is decreased reproductive capacity (Gunes *et al*., 2016). Studies on this topic often focus on the impacts of aging on female fertility, a matter assigned greater clinical relevance, and generally neglect how aging affects male fertility. This has led many to believe that aging has negligible effects on male reproductive capacity, and that men would therefore have a nearly endless reproductive lifespan. However, several studies demonstrated a direct correlation between aging and structural and functional changes of sexual organs and the endocrine system, which by their turn suggest an effect on sperm parameters and fertility (Gunes *et al*., 2016; Johnson *et al*., 2015).

Aging affects the male sexual organs in different ways. The volume of the testes starts to decrease after 60 years of age. Gonadotropin levels increase and testosterone levels decrease with aging. The number of Leydig, Sertoli, and germ cells decreases with aging. Aging introduces vascular changes that lead to testicular fibrosis. Aging has also been associated with increased incidence of benign prostatic hyperplasia, which affects ejaculation and semen volume (Mahmoud *et al*., 2003; Zitzmann, 2013; Paniagua *et al*., 1986; Neaves *et al*., 1984).

Studies show that semen parameters are negatively affected by aging (Oliveira *et al*., 2014). Decreases in semen volume caused by impaired accessory gland function, and decreased daily sperm production, total sperm count, and sperm viability have also been linked to aging (Gunes *et al*., 2016). Some authors also suggested that sperm morphology alterations occur from the age of 40 years (Neaves *et al*., 1984; Johnson *et al*., 1984).

Aging has been correlated with increased oxidative stress, which leads to increased lipid peroxidation and formation of reactive oxygen species (ROS) in the mitochondria (Gunes *et al*., 2016). The body's antioxidant capacity decreases, and oxidative damage to spermatic DNA becomes more likely. In later stages, oxidative stress may lead to cell death and decrease sperm fertilization capacity. It may also cause genetic mutations in the gametes, thereby increasing the probability of miscarriage, genetic mutations in the offspring, and metabolic, psychiatric, and neurological disorders (Kong *et al*., 2012). Male germ cells undergo continuous DNA replication and division throughout an individual's life. Therefore, older men are at greater risk of having germ cells carrying mutations (Gunes *et al*., 2016). Base substitutions account for most of the mutations and cause monogenic disorders such as achondroplasia and Apert Syndrome (Goriely & Wilkie, 2012). Hypermethylation and hypomethylation of certain DNA segments - events associated with increased incidence of schizophrenia and bipolar disorder - occur more frequently in individuals of advanced age (Goriely & Wilkie, 2012; Crow, 2000). Furthermore, telomere length generally increases with age (Eisenberg, 2011).

Considering the relevance of this topic vis-à-vis the demographic changes in progress and the insufficient attention given to it by reproductive medicine and society in general, this study aimed to identify specific changes produced by aging on semen parameters and sperm DNA integrity that might affect male fertility. The information discussed herein has use not only in the determination of couple fertility prospects, but also in the education of the general public about aging and fertility.

### MATERIALS AND METHODS

This descriptive retrospective study looked into the data from the charts of patients aged 18 years or older submitted to semen analysis at the Andrology Laboratory of Clínica las Condes in Santiago, Chile, between January 2014 and May 2017. Only the data from the more recent sample were considered for patients with more than one sample recorded in the database. The study included data from 2678 men. All samples were collected through masturbation after three to 15 days of abstinence. Sperm analysis and examination were carried out according to the techniques established by the World Health Organization (World Health Organization, 2010). Sperm DNA fragmentation was quantified via the Halosperm^®^ technique (Fernandez *et al*., 2003). Patients in abstinence for less than three or more than 15 days prior to semen analysis and individuals presenting more than 1,000,000 round cells (increased round cell count is indicative of seminal tract infection and potentially altered spermatic characteristics for causes other than age, working therefore as a confounding factor) were excluded. Included patients gave consent to joining the study before examination. The consent form had been previously approved by the institution's Ethics Committee.

The variables considered in this study were age and the following semen parameters: volume (ml); sperm concentration (millions/ml); total sperm count (millions); sperm progressive motility A+B (%); sperm morphology (% normality); and sperm DNA fragmentation (%). Normal values were defined based on the WHO standards (World Health Organization, 2010) (Table 1).

**Table 1:** Normal values for semen analysis according to the WHO*

Variable	Cut-off value
Sperm volume	>1.5ml
Sperm concentration	>15 million / ml
Total sperm count	>39 million
Sperm progressive motility (A + B)	>32%
Sperm morphology	>4%
Sperm DNA fragmentation	<30%
Non-sperm cells	<1 million / ml

* WHO laboratory manual for the examination and processing of human semen. Geneva: World Health Organization; 2010.

The patients were divided into four groups based on age. The age groups were divided as follows: 21-30 years; 31-40 years; 41-50 years; and more than 50 years.

The variables were assessed according to age using logistic regression analysis. Age was first considered a discrete quantitative variable and was later deemed a categorical variable, according to age ranges, in which individuals with ages between 21 and 30 years were set as the reference group. Odds ratios (OR) and their respective *p* values were calculated based on these analyses. Statistical significance was defined as a *p* value ≤ 0.05, and confidence intervals were set at 95%.

Correlations between age and the studied variables were searched and stratified according to the previous results and indicators mentioned above. Statistical analysis was performed on software package Stata release 15. (College Station, TX: StataCorp LLC, 2017).

### RESULTS

Our study included the data from 2678 men with ages averaging 39.2±6 years. A total of 119 individuals were aged 21-30 years; 1579 were aged 31-40 years; 852 were aged 41-50 years; and 128 were aged 50+ years. Table 2 shows the descriptive statistics for semen analysis.

**Table 2:** Semen parameters, sperm DNA fragmentation, and OR by age

Semen parameters	OR (95% CI)	*P *value
**Volume**		
31 to 40 years	0.821 (0.464 – 1.450)	0.497
41 to 50 years	1.332 (0.749 – 2.369)	0.328
Over 50 years	2.204 (1.118 – 4.334)	0.022
**Sperm concentration**		
31 to 40 years	0.987 (0.576 – 1.690)	0.962
41 to 50 years	1.188 (0.685 – 2.060)	0.538
Over 50 years	1.188 (0.685 – 2.060)	0.027
**Total sperm count**		
31 to 40 years	2.462 (0.985 – 6.150)	0.054
41 to 50 years	2.926 (1.160 – 7.381)	0.023
Over 50 years	6.151 (2.261 – 16.738)	<0.0001
**Sperm motility (A + B)**		
31 to 40 years	3.241 (1.175 – 8.940)	0.023
41 to 50 years	5.243 (1.892 – 14.526)	0.001
Over 50 years	11.911 (4.045 – 35.073)	<0.0001
**Sperm morphology**		
31 to 40 years	1.810 (0.433 – 7.556)	0.416
41 to 50 years	2.395 (0.567 – 10.119)	0.235
Over 50 years	3.223 (0.654 – 15.866)	0.150
**DNA sperm fragmentation**		
31 to 40 years	1.243 (0.364 – 4.240)	0.728
41 to 50 years	1.388 (0.399 – 4.829)	0.606
Over 50 years	4.583 (1.167 – 17.999)	0.029

OR: Odds Ratio, CI: Confidence Interval

* Parameters were dichotomized in reference to the cutoffs defined by the WHO to categorize findings as normal or anomalous.

#### Semen volume

The risk of presenting anomalous semen volumes increased with age. This finding was statistically significant among men older than 50 years (OR: 2.2; 95% CI [1.11-4.34]; *p*=0.022). Men above the age of 50 were 2.2 times more likely to present decreased semen volumes than males aged 21-30 years.

#### Sperm concentration

The risk of presenting anomalous sperm concentrations increased with age. This finding was statistically significant among men older than 50 years (OR: 2.09; 95% CI [1.08-4.02]; *p*=0.027). Men above the age of 50 were 2.09 times more likely to present anomalous sperm concentrations than males aged 21-30 years.

#### Total sperm count

The risk of presenting decreased sperm counts in semen analysis increased with age. This finding became statistically significant from 41 years of age. Men aged 41-50 years were 2.92 times more likely to present decreased sperm counts than males aged 21-30 years (OR: 2.92; 95% CI [1.16-7.38]; *p*=0.023). Men above the age of 50 were 6.15 times more likely to present decreased sperm counts than males aged 21-30 years (OR: 6.15; 95% CI [2.26-16.73]; *p*=0.0001).

#### Sperm progressive motility (A + B)

Progressive motility decreased as age increased. All age ranges presented statistically significant impairments, unlike the rest of the examined parameters. Group aged 31-40 years - OR: 3.24; 95% CI [1.17-8.94]; *p*=0.023; group aged 41-50 years - OR: 5.24; 95% CI [1.89-14.52]; *p*=0.001; group aged 50 or older - OR: 11.91; 95% CI [4.04-35.07]; *p*<0.0001; all comparisons *versus* the group aged 21-30 years.

#### Sperm morphology

No statistically significant correlation was found when patients were analyzed for age *versus* sperm morphology.

#### Sperm DNA fragmentation

The risk of presenting anomalous levels of DNA fragmentation increased with age. This finding was statistically significant among men older than 50 years old (OR: 4.58; 95% CI [1.16-17.99]; *p*=0.029). Men above the age of 50 were 4.58 times more likely to present sperm DNA fragmentation than males aged 21-30 years.

Tables 3, 4 and 5 show the descriptive statistics for sperm volume, concentration, and motility at a constant semen volume.

**Table 3:** Change in semen parameters at a constant semen volume

Sperm motility	OR (95% CI)	*p* value
**Age**	1.060 (1.038 – 1.083)	<0.0001
		
**Sperm concentration**	**OR (95% CI)**	***p* value**
**Age**	1.030 (1.010 – 1.050)	0.003

OR: Odds Ratio,

CI: Confidence Interval

**Table 4:** Change in semen parameters at a constant sperm concentration

Semen volume	OR (95% CI)	*p* value
**Age**	1.062 (1.041 – 1.084)	< 0.0001
		
**Sperm motility**	**OR (95% CI)**	***p* value**
**Age**	1.073 (1.049 – 1.098)	< 0.0001

OR: Odds Ratio,

CI: Confidence Interval.

**Table 5:** Change in semen parameters at constant sperm motility

Semen volume	OR (95% CI )	*p* value
**Age**	1.054 (1.031 – 1.078)	<0.0001
		
**Sperm concentration**	**OR (95% CI )**	***p* value**
**Age**	0.992 (0.957 – 1.027)	0.665

OR: Odds Ratio,

CI: Confidence Interval.

##### Motility and sperm concentration at a constant semen volume

Table 3 shows that, at a constant semen volume, motility decreased as age increased (OR: 1.06; 95% CI [1.03-1.08]; *p*<0.0001). Sperm concentration also decreased as age increased (OR: 1.03; 95% CI [1.01-1.05]; *p*=0.003). Therefore, each year the risk of presenting motility disorders increased 1.06 time over the previous year. As for sperm concentration at a constant volume, the risk of presenting decreased sperm concentration increased by 1.03 time over the previous year.

##### Semen volume and sperm motility at a constant sperm concentration

Table 4 shows that, at a constant sperm concentration, semen volume decreased as age increased (OR: 1.06; 95% CI [1.04-1.08]; *p*<0.0001). Sperm motility also decreased as age increased (OR: 1.07; 95% CI [1.04-1.09]; *p*<0.0001). Therefore, each year the risk of presenting lower semen volumes increased 1.06 time over the previous year when concentration was kept constant. The risk of presenting impaired sperm motility increased 1.07 time each year when concentration was kept constant.

##### Sperm volume and concentration at constant motility

Table 5 shows that semen volume decreased as age increased when motility was kept constant (OR: 1.05; 95% CI [1.03-1.07]; *p*<0.0001). Therefore, each year the risk of presenting lower semen volumes increased 1.05 time over the previous year when motility was kept constant.

#### DISCUSSION

Aging is an inevitable process associated with multiple physiological changes, some of which affect the reproductive organs. This study focused on the effects of aging on semen parameters based on routine semen analyses and percent sperm DNA fragmentation. Although sperm parameters may change with aging, alterations do not necessarily correlate with male fertility outcomes. Nonetheless, other authors have attempted to find correlations between age and outcomes of assisted reproduction procedures (Das *et al*., 2013).

Tables 2, 3, 4 and 5 show the different semen parameters analyzed in our study. The tables show the relationships between age and semen parameters, describe the links between them, and report the risks assigned to them, which were combined to yield objective associations. Our findings suggested that semen volume, sperm concentration, total sperm count, and sperm motility correlated negatively with age. When compared to males aged 21-30 years, men above the age of 50 were 2.2, 2.09, 6.15, and 11.91 times more likely to present lower semen volumes, lower sperm concentration, lower total sperm count, and impaired sperm progressive motility, respectively. Our findings were consistent with the data reported by Verón *et al*. (2018), thus confirming the effect of aging on semen parameters. They were also similar to the findings described by Stone *et al*. (2013), in which negative correlations between age and semen volume, sperm concentration, and progressive motility were derived. Although Oliveira *et al*. (2014) reported an association between age and semen volume and sperm progressive motility, the authors were unable to find a correlation between age and sperm concentration. However, Harris *et al*. (2011) emphasized the importance of sperm concentration and how spermatid concentration in seminiferous tubules decreased with age, thus accounting for the reduction of sperm concentration as age advances.

Sperm motility seems to be the parameter more significantly affected by age, since individuals in all age ranges presented a significantly increased risk of having anomalous findings compared to males aged 21-30 years. Spermatozoa acquire motility in the prostate and epididymis. Therefore, the impairment observed with aging might be explained by the gradual decline individuals experience in endocrine function as they age. Harris *et al*. (2011) concluded that motility is affected with aging, in annual decreases of 0.17-0.8%, which result in 3-16% decreases in motility over 20 years.

Differently from other authors (Neaves *et al*., 1984; Johnson *et al*., 1984; Harris *et al*., 2011), we were unable to find a statistically significant association between sperm morphology and age. Interestingly, Harris *et al*. (2011) reported that the most significant changes in semen quality are oligospermia, asthenospermia, and teratospermia, suggesting a progressive decline in normal sperm morphology equivalent to 0.2-0.9% per year of age. Other authors have reported that over a 20-year period 4-18% of the spermatozoa present altered morphology (Auger *et al*., 1995; Andolz *et al*., 1999; Bujan *et al*., 1988). The disagreement between our findings and the studies of other authors might stem from changes in morphology assessment over the last few decades, disparities between study designs, or differences in statistical analysis.

Our study found a correlation between age and sperm DNA fragmentation. Males above the age of 50 presented a statistically significant increase in DNA damage and were 4.58 times more likely to present sperm DNA fragmentation than men aged 21-30. Other authors using the same technique described similar findings (Varshini *et al*., 2012; Oliveira *et al*., 2014). Petersen *et al*. (2018) also concluded that DNA fragmentation worsened with age and associated it with mitochondrial damage, since mitochondrial membrane potentials deteriorate significantly with age. One might argue that it is advisable to assess older men seeking fertility treatment for DNA fragmentation, since it may cause infertility (Ahmadi *et al*., 2016) and increase miscarriage rates (García-Ferreyra *et al*., 2015). Antioxidant therapy might be an option to treat men with sperm damage (Ahmadi *et al*., 2016).

When three variables were correlated (sperm concentration, volume, and motility), our data agreed with the previous analysis and showed increased semen parameter impairment with aging. A point to consider is that the data used in our study were collected from the male partners of infertile couples, a factor that might have introduced some selection bias. Our patients had been scheduled to undergo semen analysis and might represent a reproductively compromised population when compared to other men in general. In addition, our study did not control for other variables such as obesity, smoking, or alcohol drinking. These variables may also affect sperm parameters (García-Ferreyra *et al*., 2015; Oliveira *et al*., 2018). More studies with greater numbers of normal fertile individuals must be performed to confirm the effects of aging on sperm concentration, total sperm count, sperm motility, and sperm DNA fragmentation. Nevertheless, the findings reported in this and other similar studies (Varshini *et al*., 2012; Stone *et al*., 2013; Oliveira *et al*., 2014; Gunes *et al*., 2016; Petersen *et al*., 2018) suggest that aging has a negative effect on most semen parameters and DNA integrity. Therefore, age is an important factor to consider in the treatment of infertile couples.
